# Effectiveness of transdermal fluralaner in the treatment of *Lynxacarus radovskyi* (Acari: Listrophoridae) in naturally infested domestic cats

**DOI:** 10.1590/S1984-29612023067

**Published:** 2023-11-27

**Authors:** Bárbara Monteiro Coimbra Guimarães, Rafaella Tortoriello, Luísa Xavier Christ, Camila Sampaio Martins Land Manier, Diefrey Ribeiro Campos, Luciano da Silva Alonso, Julio Israel Fernandes

**Affiliations:** 1 Programa de Pós-graduação, Instituto de Veterinária, Universidade Federal Rural do Rio de Janeiro – UFRRJ, Seropédica, RJ, Brasil; 2 Departamento de Parasitologia Animal, Instituto de Veterinária, Universidade Federal Rural do Rio de Janeiro – UFRRJ, Seropédica, RJ, Brasil; 3 Departamento de Medicina Veterinária e Cirurgia, Instituto de Veterinária, Universidade Federal Rural do Rio de Janeiro – UFRRJ, Seropédica, RJ, Brasil

**Keywords:** Lynxacarus radovskyi, fluralaner, ectoparasiticide, Lynxacarus radovskyi, fluralaner, ectoparasiticida

## Abstract

Mites of the species *Lynxacarus radovskyi*, which are commonly found on domestic cats in Brazil, can cause discomfort, itching, and alopecia. The development of new, safer and more effective treatments with a broad spectrum of activity, including the use of isoxazolines, is needed. The purpose of this study was to assess the efficacy of transdermal fluralaner in domestic cats naturally infested with *L. radovskyi*. Twenty cats were evaluated by trichograms and divided into two groups of 10 animals. The control group was not treated, while the treated group was given a single topical dose of fluralaner, as per the manufacturer’s instructions. The cats were reassessed for the presence of *L. radovskyi* eggs and mites on days D+7, D+14, D+28, D+42, D+56, D+70, D+84, and D+98. As of D+42, all the animals (100%) tested negative for mites, and remained parasite-free until the end of the study, while the control group tested positive throughout the experiment. It can be concluded that a single dose of fluralaner applied topically was effective in treating cats naturally infested with *L. radovskyi*.

## Resumo

Os ácaros da espécie *Lynxacarus radovskyi*, comumente encontrados em gatos domésticos, no Brasil, podem causar desconforto, prurido e alopecia. É necessário o desenvolvimento de novos tratamentos mais seguros e eficazes com amplo espectro de ação, incluindo o uso de isoxazolinas. O objetivo deste estudo foi avaliar a eficácia do fluralaner transdérmico em gatos domésticos naturalmente infestados com *L. radovskyi*. Vinte gatos foram avaliados por tricogramas e divididos em dois grupos de 10 animais. O grupo controle não foi tratado, enquanto o grupo tratado recebeu uma única dose tópica de fluralaner, conforme instruções do fabricante. Os gatos foram reavaliados quanto à presença de ovos e ácaros de *L. radovskyi* nos dias D+7, D+14, D+28, D+42, D+56, D+70, D+84 e D+98. A partir de D+42, todos os animais (100%) testaram negativo para ácaros e permaneceram livres do parasita até o final do estudo, enquanto o grupo controle apresentou resultado positivo durante todo o experimento. Pode-se concluir que uma única dose de fluralaner aplicada topicamente foi eficaz no tratamento de gatos naturalmente infestados com *L. radovskyi*.

## Introduction

*Lynxacarus radovskyi* Tenório, 1974 (Sarcoptiformes, Listrophoridae) is the mite species that causes a parasitic dermatosis known as lynx acariasis. These mites, which are parasites that infest the fur of domestic and wild felids, occur mostly in tropical and humid climates. The mite’s life cycle begins on the host, through direct contact or via fomites (Rocha et al., 2019).

Most infected animals are asymptomatic. However, some cats may have miliary dermatitis, hypotrichosis, and itching (Clare et al., 2004; Han et al., 2016), which are not related to the degree of parasitism (Ketzis et al., 2016).

The parasitological diagnosis is made after identifying the mites attached to the fur with a magnifying glass, which is made easier by the “salt and pepper” appearance of the animal’s hairs (Clare et al., 2004), or even a trichogram by means of a stereoscopic microscope to identify the mites attached to the hair shafts (Ketzis et al., 2016).

The control of this mite is relatively simple, but research has focused on developing safer and more effective drugs with a broader spectrum of activity, such as isoxazolines (Zhou et al., 2022), that are easy to administer, particularly in the case of felines (Campos et al., 2021). This study focused on ascertaining the efficacy of a single dose of topically administered fluralaner (Bravecto ™) for the treatment of lynx acariasis in naturally infested domestic cats.

## Materials and Methods

The entire study was conducted according to the best clinical practices outlined in the guidelines of the American Association of Feline Practitioners and the International Society of Feline Medicine (Rodan et al., 2011).

Twenty domiciled cats were naturally infected by *L. radovskyi* mite, as determined by trichoscopy, and confirmed visually under an optical microscope, were chosen for the study. These cats had not been exposed to any insecticides or acaricides for a period of 90 days prior to the study.

The animals were randomly divided into two groups of 10. The animals in the control group were not treated, while those in the treated group were given a single dose of fluralaner topical solution, as recommended by the manufacturer.

The methodology used to determine efficacy was the same as that described by Han et al. (2016). For a total of nine samples per cat per day of evaluation, three trichograms were conducted, each involving the removal of about 50 hairs from three predetermined areas: the dorsal region of the neck, lateral lumbar region, and perineal region and/or tail. Samples were collected on day 0 (treatment day) and days D+7, D+14, D+28, D+42, D+56, D+70, D+84, and D+98.

The collected hair samples were placed between slides and coverslips and examined under an optical microscope. The animals were given scores based on the number of eggs/mites recovered: 0, when no mites or eggs were found in any sample; 1, only non-adhering eggs were found; 2, only adhering eggs were found; 3, ≤50 mites were seen; 4, >50 mites were seen. The final score was determined by adding up the scores found in the nine trichograms.

The difference between the mean values of the control and treated animals was used to calculate the acaricidal efficacy, using a formula adapted from Abbott (1925). Efficacy (%) = 100 × (mean of control group scores – mean of treated group scores / mean of control group scores). The data were tabulated for statistical analysis, and the medians of the pre- and post-treatment scores between the groups were compared using the Mann–Whitney test, with a significance level of 5%. The statistical software BiosEstat 5.3 was used for all the calculations.

## Results

The 20 domestic cats involved in this study were all classified as mixed breed and domiciled. The animals ranged in age from 2 to 9 years and weighed between 2 and 5 kg.

Table 1 lists the scores obtained after conducting the trichograms, the respective means, and the medians over the experimental days. On day zero (D0), the experimental groups had similar mean infestations and showed no statistically significant difference (p = 0.3258), indicating that the groups were distributed homogeneously. All animals of the control group remained untreated, i.e., parasitized, during the study.

**Table 1:** Mean and median parasitological scores of domestic cats in the control groups and those treated with topical fluralaner applied in a single dose, including efficacy, in domestic cats naturally infested with *Lynxacarus radovskyi*.

	**Experimental Day**								
	**D0**	**D7**	**D14**	**D28**	**D42**	**D56**	**D70**	**D84**	**D98**
Control group									
Number of positive animals	10	10	10	10	10	10	10	10	10
Mite score (min - max)	12 -25	12 - 22	7 -23	5 - 26	9 -19	9 - 24	8 - 20	8 - 20	7 - 17
Mediana	18	16	18	15	13.5	14	12	14	13.5
Mean (standart derivation)	19.1 (4.8)	16.2 (2.9)	16.5 (5.6)	16.4 (6.3)	13.8 (3.4)	15.4 (5.0)	13.4 (4.6)	13.3 (4.4)	12.5 (3.4)
Treated group									
Number of positive animals	10	9	8	2	0	0	0	0	0
Mite score (min - max)	(8 - 31)	(0 - 20)	(0 - 10)	(0 - 3)	0	0	0	0	0
Mediana	17.5	4	2	0	0	0	0	0	0
Mean (standart derivation)	17.6 (7.2)	5.6 (5.7)	3 (3.2)	0.4 (1.0)	0	0	0	0	0
Efficacy	- - -	65.4	81.8	97.6	100.0	100.0	100.0	100.0	100.0
p-value	0.3258	0.0017	0.0002	0.0002	0.0002	0.0002	0.0002	0.0002	0.0002

Statistical analysis performed using the Mann–Whitney test.

The efficacy rate in controlling *L. radovskyi* mites observed on D+7, D+14, and D+28 was 64.5%, 81.8%, and 97.6%, respectively. It should be noted that on day 7, the groups showed statistical differences and on day D+42, the effectiveness was 100%, remaining unchanged until D+98, the last day of the animal evaluation.

None of the 10 cats topically treated with transdermal fluralaner exhibited any adverse effects from the medication and, none of the animals in this study exhibited these clinical signs.

## Discussion

Although other drugs such as ivermectin (Foley, 1991), fipronil (Clare et al., 2004) and d-phenothrin associated with pyriproxyfen (Souza et al., 2012) have been described as effective in the treatment of lynx acariasis in cats, some drugs must be used cautiously in view of possible adverse reactions (Boland & Angles, 2010). Han et al. (2016) combined moxidectin and imidacloprid, but two doses administered at a 15-day interval were required for effectiveness. In this context, safer, more effective molecules with a broad spectrum of action are needed. In the current study, none of the animals treated with transdermal fluralaner suffered any visible side effects.

A new class of ectoparasiticides, the isoxazolines, have been widely used to control ectoparasites in dogs, but their use in cats is limited. These drugs are often used off-label or are prescribed for certain parasites, but not for *L. radovskyi* mites, which were treated in this study. In Brazil and other countries, the product administered for this treatment is registered only for flea control (Ranjan et al., 2018), although it has been employed topically to control *Otodectes cynotis* (Hering, 1838) (Taenzler et al., 2017), *Sarcoptes scabiei* (Linnaeus, 1758) (Curtis et al., 2019), and *Dermatobia hominis* (Linnaeus, 1781) (Campos et al., 2021). According to the authors, this is the first field study that addresses the efficacy of topical fluralaner administered for the treatment of lynx acariasis. Fluralaner has already been used to control *L. radovskyi* in felines. Han et al. (2016) reported that a single oral dose of fluralaner showed 100% efficacy on D+28 of their study. The result reported by those authors is better that achieved in our study, in which 100% efficacy was observed only on day D+42. A possible explanation for this difference is that the drug was administered transdermally, which may have influenced the dispersion or concentration of the active ingredient. Fluralaner administered orally is more effective, but the practicality for owners and their cats of administering the same drug topically is a factor that veterinarians take into account when prescribing an antiparasitic (Lavan et al., 2021).

Because the animals in the current study were isolated from other infested animals, the residual effect against reinfestations as described by Han et al. (2016) could not be evaluated.

Campos et al. (2020) found that the sarolaner was effective in controlling *L. radovskyi* in domestic cats, showing >95% efficacy from day D+30 when combined with another isoxazoline. This finding is similar to that achieved by fluralaner administered orally (Han et al., 2016). In comparison to topical administration, oral administration appears to promote higher and faster efficacy. Campos et al. (2020) used a method similar to that used in this study, medicating the animals with a single dose and monitoring them until D+60. However, they reported less than 100% effectiveness, unlike the results achieved in our study, in which the cats remained free of parasites until D+98, the last day the animals were monitored. This suggests that the topical administration of fluralaner provides an additional benefit, since the effect of the medication lasts up to 12 weeks, according to Kilp et al. (2016), thus reducing the stress resulting from restraint, handling, and application of a new dose of antiparasitic.

## Conclusion

A single dose of fluralaner topical solution was effective in controlling *L. radovskyi* in naturally infested cats.
